# Toward a Better Understanding of Neuronal Migration Deficits in Autism Spectrum Disorders

**DOI:** 10.3389/fcell.2019.00205

**Published:** 2019-09-20

**Authors:** Yi-Hsuan Pan, Nan Wu, Xiao-Bing Yuan

**Affiliations:** ^1^Key Laboratory of Brain Functional Genomics (Ministry of Education and Shanghai), Institute of Brain Functional Genomics, School of Life Sciences and the Collaborative Innovation Center for Brain Science, East China Normal University, Shanghai, China; ^2^Department of Anatomy and Neurobiology, University of Maryland School of Medicine, Baltimore, MD, United States

**Keywords:** autism-spectrum disorders, neuronal migration, brain structural abnormalities, mechanism-based therapy, E/I balance

## Abstract

Newborn neurons in developing brains actively migrate from germinal zones to designated regions before being wired into functional circuits. The motility and trajectory of migrating neurons are regulated by both extracellular factors and intracellular signaling cascades. Defects in the molecular machinery of neuronal migration lead to mis-localization of affected neurons and are considered as an important etiology of multiple developmental disorders including epilepsy, dyslexia, schizophrenia (SCZ), and autism spectrum disorders (ASD). However, the mechanisms that link neuronal migration deficits to the development of these diseases remain elusive. This review focuses on neuronal migration deficits in ASD. From a translational perspective, we discuss (1) whether neuronal migration deficits are general neuropathological characteristics of ASD; (2) how the phenotypic heterogeneity of neuronal migration disorders is generated; (3) how neuronal migration deficits lead to functional defects of brain circuits; and (4) how therapeutic intervention of neuronal migration deficits can be a potential treatment for ASD.

## Introduction

Autism spectrum disorders (ASD) are heterogeneous developmental disorders with both strong genetic bases and environmental influences. Core symptoms of ASD include poor social skills, impaired language abilities, and restricted interests or repetitive/stereotyped behaviors. These symptoms emerge during early postnatal development of autistic children (Dietert et al., 2011; Chaste and Leboyer, 2012; Jeste and Geschwind, 2014). The cellular etiology of ASD involves one or multiple abnormalities of developmental events including neurogenesis, neuronal migration, axon projection, dendrite development, spine formation, synaptogenesis, and synapse remodeling. It is generally believed that reduced GABAergic inhibitory tone occurs in autistic brains (Ferguson and Gao, 2018) resulting in altered excitation/inhibition (E/I) balance of ASD-relevant brain circuits (Bozzi et al., 2018; Ferguson and Gao, 2018). Currently, there is no effective treatment for ASD, primarily because of poor understanding of how genetic defects and environmental factors alter the structure and function of relevant brain circuits.

Neuronal migration is a fundamental event in the development of brain circuits. After being produced in germinal zones, young neurons actively migrate to designated brain regions to be wired into functional circuits. The motility and trajectory of migrating neurons are regulated by both extracellular factors and intracellular signaling cascades (Hatanaka et al., 2016). Neuronal migration in developing brains is highly sensitive to various physical, chemical, and biological insults as well as genetic mutations. Defects in the molecular machinery of neuronal migration lead to mis-localization of affected neurons. The consequent malformation and malfunction of various brain circuits are considered as an important etiology of multiple neurodevelopmental disorders including epilepsy, intellectual disability, dyslexia, schizophrenia, and ASD (Galaburda et al., 1985; Chang et al., 2005; Guerrini, 2005; Alexandre et al., 2006; Wegiel et al., 2010). In addition to neuronal migration, genetic and environmental factors also affect other developmental processes, including axon growth and pathfinding, dendrite arborization and pruning, spine morphogenesis, synapse formation and remodeling. In current studies of neurodevelopmental disorders, much attention has been paid to the role of risk factors in synapse formation and remodeling. The relationship between neuronal migration deficits and functional defects of brain circuits, however, has been largely ignored. For mechanism-based therapy of neurodevelopmental disorders, it is important to clarify whether neuronal migration deficits are general characteristics of the disease and how aberrant neuronal migration leads to defects in the function of disease-relevant brain circuits. This review focuses on neuronal migration deficits in ASD. In addition to reviewing clinical, neuropathological, and experimental evidence of neuronal migration deficits in ASD, we, from a translational perspective, also discuss how prevalent neuronal migration deficits occur in autistic brains, how the heterogeneity of disease phenotype is generated, and how aberrant neuronal migration is mechanistically linked to brain circuit deficits. The potential of treating ASD associated with neuronal migration deficits by restoring neuronal migration is also discussed.

## Are Neuronal Migration Deficits General Neuropathological Characteristics of ASD?

### Deformation of Autistic Brains Due to Aberrant Neuronal Migration

From the perspective of ASD treatment, it is important to determine whether neuronal migration deficits are a general feature of ASD. Various forms of brain malformations in some autistic individuals have been frequently discovered by magnetic resonance imaging (MRI), including altered brain volume, specifically the volume of cerebellum, frontal lobe, and limbic system; enlarged brain ventricles; reduced cortex thickness; various types of heterotopia; abnormal cortical gyrification; and abnormal white matter volume (Acosta and Pearl, 2004; Ke et al., 2008; Scott et al., 2009; Nickl-Jockschat et al., 2012; Yang et al., 2016). Reduced volume of certain brain regions had been considered as an evidence of neuronal migration deficits in ASD (Peterson, 1995). However, since other developmental processes, including the proliferation of neural progenitor cells, generation of neurons and glial cells, neurite growth and pruning, and cell survival, also directly affect the brain volume, MRI findings of volumetric changes of ASD-associated brain regions are not a direct indication of abnormal neuronal migration. MRI-detectable brain structural abnormalities that are more directly related to neuronal migration deficits include lissencephaly and various types of heterotopias. In developing cerebral cortex, hippocampus, and cerebellum of mammals, neuronal migration guided by radial glial fibers (radial migration) is essential for the formation of the laminar architecture and ultimately the proper patterning of synaptic connectivity of neurons (Hatten, 2002). Abnormal radial migration is a major cause of structural abnormalities in these brain regions. Cases of ASD associated with pachygyria, dysplasia, and heterotopia have been frequently reported (Korkmaz et al., 2006; Beal, 2014; Zare et al., 2019), implying abnormal radial migration in these individuals.

It remains unclear how frequent these structural abnormalities occur in ASD and in typically developing peers due to the lack of a systematic comparison and consistency in clinical imaging studies. However, several small scale studies have revealed a frequency of 30 – 50% in ASD (Table 1). One MRI study reported cortical malformations in 7 of 13 high-functioning male autistic subjects, including polymicrogyria in 5 subjects, schizencephaly and macrogyria in 1 subject, and macrogyria in 1 subject. None of the 13 matched controls displayed any cortical abnormalities. These structural abnormalities of the cortex are believed to arise from deficits in radial migration of cortical neurons during the first 6 months of gestation (Piven et al., 1990). A study combining positron emission tomography (PET) and MRI in 13 infantile autistic individuals showed that 5 of 13 autistic subjects displayed neuronal migration abnormalities (focal pachygyria) (Schifter et al., 1994). Postmortem MRI analyses of brains from 13 autistic and 14 control subjects revealed subcortical, periventricular, hippocampal, and cerebellar heterotopias in 4 autistic subjects (Wegiel et al., 2010). The percentages of autistic individuals with MRI-detectable structural abnormalities caused by abnormal neuronal migration are highly variable in these studies, possibly due to the difference in patient selection, imaging resolution, and diagnose standard.

**TABLE 1:** Percentages of ASD patients with diagnosable structural abnormalities of the brain in MRI studies.

**MRI studies**	**Patient selection**	**ASD subjects**				**Control subjects**			
			
		**Total no.**	**Gender**	**Structural abnormalities relevant to neuronal migration deficits**	**%**	**Total no.**	**Gender**	**Structural abnormalities relevant to neuronal migration deficits**	**%**
Piven et al., 1990	High-functioning autistic	13	M (13)	Polymicrogyria (5) Schizencephaly and macrogyria (1) Macrogyria (1)	53.8	13	M (13)	0	0
Schifter et al., 1994	Infantile autistic	13	M (9) F (4)	Focal pachygyria (5)	38.5	–	–	–	–
Wegiel et al., 2010	Autistic	13	M (9) F (4)	Subcortical heterotopia (2) Periventricular heterotopia (1) Hippocampal heterotopia (1) Cerebellar heterotopia (1)	30.8	14	M (9) F (5)	0	0

*M, male; F, female; %, percentage of subjects with structural abnormalities relevant to neuronal migration deficits; Numbers in brackets, numbers of subjects of each category; Red, co-occurred with one of the above phenotypes; –, not available.*

Neuropathological analysis of postmortem brain tissues allows characterization of brain cytoarchitecture at a much higher resolution to reveal mild structural abnormalities that cannot be detected by clinical MRI. Qualitative and quantitative analysis of postmortem brain sections showed altered laminar distribution of neurons, poor patterning of the gray–white matter boundary, and supernumerary neurons in the layer I or white matter in most ASD cases (Bailey et al., 1998; Hutsler et al., 2007; Simms et al., 2009; Avino and Hutsler, 2010; Oblak et al., 2011). As summarized in Table 2, a clinicopathological study showed that 4 of 6 autistic brains displayed disturbed neuronal lamination in various regions of the cerebral cortex and ectopic distribution of neurons in the white matter (Bailey et al., 1998). In a systematic analysis of whole brain serial sections, unusually small soma size, more closely compacted neurons, and less distinctive laminar architecture were discovered in the anterior cingulate gyrus in 8 of 9 autistic brains (Kemper and Bauman, 1998). A stereological analysis of the anterior cingulate cortex showed irregular lamination in 3 of 9 autistic brains and increased density of neurons in the subcortical white matter in the remaining cases (Simms et al., 2009). A follow up study by the same research group reported altered neuronal distribution in different layers and increased neuronal number in the white matter of posterior cingulate cortex in all 8 autistic brains (Oblak et al., 2011). A recent small scale postmortem study reported that focal disruption of cortical laminar architecture occurred in prefrontal and temporal cortex in 10 of 11 children with ASD and 1 of 11 unaffected children (Stoner et al., 2014). These alterations in cortical cytoarchitecture in autistic brains are strong indication of defects in the radial migration of cortical neurons during development, although failed neuronal apoptosis could also result in increased neuronal density in the white matter and layer I of the cortex (Avino and Hutsler, 2010). Together with the aforementioned MRI imaging studies, these neuropathological findings support the notion that most, if not all, individuals with ASD have defects in neuronal migration in certain regions of the brain, although in most ASD individuals, neuronal migration deficits are mild and the overall cytoarchitecture of the brain is maintained (Hutsler et al., 2007).

**TABLE 2:** Percentages of autistic brains with aberrant neuronal migration revealed by neuropathological studies.

**Neuropathological studies**	**ASD subjects**					**Control subjects**				
		
	**Total no.**	**Gender**	**Aberrant neuronal migration**	**Affected brain area**	**%**	**Total no.**	**Gender**	**Aberrant neuronal migration**	**Affected brain area**	**%**
Bailey et al., 1998	6	M (5) F (1)	Disturbed neuronal lamination (4)	Frontal cortex	66.7	7	M (5) F (2)	–	–	–
Kemper and Bauman, 1998	9	–	Less distinctive laminar architecture (8)	Anterior cingulate cortex	88.9	9	–	–	–	–
Simms et al., 2009	9	M (9)	Irregular lamination (3) increased neurons in WM (6)	Anterior cingulate cortex	100	4	M (4)	0	–	0
Oblak et al., 2011	8	M (8)	Increased neurons in WM (8)	Posterior cingulate cortex	100	8	M (6) F (2)	–	–	–
Stoner et al., 2014	11	M (8) F (3)	Disruption of cortical laminar architecture (10)	Prefrontal and temporal cortex	90.9	11	M (8) F (3)	Disruption of cortical laminar architecture (1)	Prefrontal and temporal cortex	9.1

*WM, white matter; M, male; F, female; %, percentage of brains with aberrant neuronal migration; Numbers in brackets, numbers of subjects of each category; –, not described/available.*

Structural MRI and neuropathological findings also showed that neuronal migration deficits in autistic brains are highly heterogeneous with respect to locations of occurrence, affected cell types, quantity of ectopic neurons, and the associated structural changes. Aberrant neuronal migration in autistic subjects or rodent ASD models has been observed in most brain regions relevant to autistic behaviors, including the frontal and temporal cortex, the cingulate cortex, the cerebellum, and the raphe nuclei. In the cerebral cortex, deficits in both radial migration of pyramidal neurons (Fukuda and Yanagi, 2017) and tangential migration of GABAergic interneurons (Provenzano et al., 2017) have been observed in subsets of individuals with ASD. In the cerebellum of autistic brains, the presence within the dysplastic nodule of both GABAergic Purkinje cells produced from the cerebellar ventricular zone and glutamatergic granule neurons produced from the rhombic lip indicates abnormal migration of these two major cell types (Wegiel et al., 2010). In the raphe nuclei, defects in the migration of serotonin (5-hydroxytryptamine [5-HT]) neurons may cause abnormal 5-HT levels in the prefrontal cortex of autistic brains (Miyazaki et al., 2005; Kuwagata et al., 2009).

Deficits in neuronal migration have also been observed in many rodent genetic models of ASD that mimic disease-causing mutations in autistic individuals (Penagarikano et al., 2011; Feliciano et al., 2012; Provenzano et al., 2017). In rodent models that mimic ASD induced by environmental factors such as maternal immunization (Patterson, 2011), prenatal exposure to valproic acid (VPA) (Ranger and Ellenbroek, 2016), and perinatal exposure to glufosinate ammonium herbicide (Herzine et al., 2016), neuronal migration deficits are prominent features (Kuwagata et al., 2009; Oskvig et al., 2012).

### Neuronal Migration Deficits in Autistic Brains Are Underdiagnosed

As mentioned above, more than 30% of ASD patients have clinical manifestation of structural brain malformations that reflect neuronal migration deficits (Table 1). As mild migration deficits may be beyond the detection capacity of clinical imaging technologies (Schumann and Nordahl, 2011), the involvement of neuronal migration deficits in ASD without visible brain deformation cannot be completely ruled out. Clinical manifestation of brain malformation depends on the accumulation of a large number of ectopic neurons in a focal area. If a small population of ectopic neurons scatter in a broad area of the brain, the general tissue structure would still be maintained albeit the existence of misplaced neurons. Under such situation, ectopic neurons can only be revealed by histological analysis of postmortem brain tissues. In most ASD individuals, neuronal migration deficits are mild and do not cause severe brain structural abnormalities. The pathogenic impact of mild neuronal migration deficits should not be ignored, because studies in animals have shown that even very subtle migration deficits involving only ten thousands of cortical neurons caused long-lasting behavioral abnormalities (Vomund et al., 2017). Another example is that activation of the mTOR pathway in the interneuron lineage disrupts interneuron migration to the cortex and reduces the seizure threshold, although this does not lead to a visible abnormality of the brain (Fu et al., 2012; D’Gama et al., 2017). A more rigorous method for detecting this type of diffused neuronal heterotopia is to specifically label different subpopulations of neurons in postmortem brain tissues. Such technique has not been widely applied in clinical pathology analysis, although it has been a laboratory routine in analyzing animal tissues.

Some ectopic neurons undergo apoptosis due to insufficient trophic support from the brain area of the migration destination. These neurons are eliminated at later developmental stages, resulting in a negative diagnosis. This possibility is supported by findings in animal studies. *In utero* electroporation of embryonic cortical neurons with shRNAs against the ASD risk gene Robo4 in mouse and rat led to deficits in radial migration of cortical neurons, and the total number of neurons mis-localized in deep cortical layers was markedly reduced at young adult stage compared to neonatal stage (Zheng et al., 2012). A similar study conducted in rat embryos also showed a loss of neurons that did not migrate properly after knockdown of the disabled-1 (Dab1) gene (Vomund et al., 2017).

Taken together, neuronal migration deficits represent an important pathological feature of autistic brains, although some individuals with ASD lack clear clinical diagnosis of neuronal migration deficits. Due to the inability of current clinical imaging techniques in detecting diffused lesions, the percentage of autistic subjects with disorganized neuronal migration may have been underestimated in clinical reports. Neuronal migration deficits should be an important biomarker for ASD and an intervention target for its treatment.

## Mechanisms Underlying the Phenotypic Heterogeneity of Neuronal Migration Deficits

Theoretically, behavioral consequence of neuronal migration deficits depends on the brain region that has lost proper input or output connectivity due to abnormal migration of a group of neurons. Genetic studies have revealed a group of risk genes for multiple psychiatric diseases (Cross-Disorder Group of the Psychiatric Genomics Consortium, 2013b). Moreover, mutations in a single ASD risk gene may be associated with different degrees of social disability and different comorbid disorders including schizophrenia, intellectual disability, language impairment, epilepsy, and delayed development (Cross-Disorder Group of the Psychiatric Genomics Consortium, 2013a). One potential reason why mutations in a single gene can cause a broad spectrum of clinical manifestations is that most ASD risk genes are large genes with multiple functional domains, each interacting with a specific signaling complex. Mutations in different domains of the same molecule may lead to different or even opposite impact on its downstream signaling cascades. Therefore, the phenotypic consequences of mutations of a single gene at different sites may be substantially different.

Recently, somatic mutations have emerged as another important mechanism for neuronal migration disorders with or without visible brain lesions. These mutations contribute to a broad range of neuropsychiatric conditions including epileptic encephalopathies, intellectual disability, and ASD (D’Gama and Walsh, 2018). Unlike traditional genetic mutations that are inherited from germline cells and are present in all cells of an affected individual, somatic mutations occur postzygotically and are present in a subset of somatic cells. Human brain is found to be highly vulnerable to somatic mutations during the period of active neurogenesis (Lim et al., 2017; D’Gama and Walsh, 2018). It has been shown that a low level of brain somatic mutations is sufficient to cause intractable focal epilepsy (Lim et al., 2015; Nakashima et al., 2015). Somatic mutations in neuronal migration-associated genes randomly occur in different groups of neural progenitors or at different developmental stages, leading to different types of focal heterotopia (Pilz et al., 1999; Quelin et al., 2012; Blumcke and Sarnat, 2016). Therefore, identical mutations in the same neuronal migration-related gene may result in heterogeneous disease phenotypes depending on when the mutation occurs and what subpopulations of neurons are hit.

## Association of Neuronal Migration Deficits with Compromised Network Functions

Due to the pleotropic nature of most genes, defects in brain network functions upon gene mutations usually involve multiple mechanisms. Some gene mutations directly affect electrophysiological properties and synaptic transmission of neurons in brain circuits, while others may indirectly alter network activity by affecting various early developmental processes, including neurogenesis, neuronal migration, and axon/dendrite development. Defects in these early developmental processes have a wide range of consequences for a timely, coordinated establishment of interconnected neuronal networks. Based on findings from clinical neuropathology and animal model studies, we propose several potential mechanisms of how abnormal neuronal migration may affect the wiring and function of brain circuits.

One possibility is that compromised network function is caused by abnormal function of misplaced neurons due to the lack of appropriate afferent innervation at an ectopic location. Many axons in the brain travel and innervate target cells in a lamina-specific pattern (Huberman et al., 2010). Although the overall laminar structure is maintained when a small number of neurons fail to migrate to the proper cell layer, mis-located neurons may lose the innervation by fibers that travel and innervate neurons in the designated cell layer and thus do not fire properly. Consistent with this notion is the finding that deficits in the radial migration of cortical neurons induced by *in utero* electroporation of rat cortex with shRNAs against the doublecortin (Dcx) gene severely reduced both glutamatergic and GABAergic synapses in ectopic neurons (Ackman et al., 2009; Martineau et al., 2018).

Another possibility is that the failure of ectopic neurons to innervate proper targets leads to abnormal firing of target neurons. A typical example of such scenario is that the failure of cortical interneurons to invade and populate the cortex resulted in reduced inhibition of the cortical network. The distal-less (Dlx) homeobox genes Dlx1, 2, 5, and 6 play important roles in the maturation and migration of developing GABAergic interneurons (Anderson et al., 1997). Point mutations in Dlx1, 2, and 6 genes have been detected in ASD patients (Liu et al., 2009; Nakashima et al., 2010), implicating a role of GABAergic dysfunction in ASD. Dlx1 and 2 are required for the proper migration of all GABAergic interneurons, and Dlx5 and 6 are required for the development of MGE-derived parvalbumin-expressing interneurons that populate the cortex and hippocampus (Wang et al., 2010). In Dlx1/2 double knock-out mice, tangential migration of interneurons from ganglionic eminences to the cortex and hippocampus is completely abolished (Anderson et al., 1997; Cobos et al., 2005). In Dlx1 or Dlx2 single knock-out mice, interneuron migration is generally intact, but they have fewer interneurons, abnormal neurite morphology, and increased seizure susceptibility (Cobos et al., 2005, 2007; Mao et al., 2009). Misplaced cortical pyramidal neurons due to deficits in radial migration are able to project their axons to appropriate target regions (D’Amato and Hicks, 1980; Jensen and Killackey, 1984; Lee et al., 1997; Ackman et al., 2009). However, recent studies combining diffusion tensor imaging and resting state functional MRI revealed abnormal structural and functional connection between heterotopia and overlaying gray matter in patients with periventricular nodular heterotopia (PVNH) (Christodoulou et al., 2012; Liu et al., 2017). Electrophysiology studies of cortical slice from multiple rat models of PVNH showed that the overlaying normotopic gray matter generates epileptiform discharge that propagates to the heterotopic gray matter, suggesting that aberrant migration of heterotopic neurons triggered plastic changes of the overlaying normotopic neurons to cause seizures (Sancini et al., 1998; Zhu and Roper, 2000; Benardete and Kriegstein, 2002; Ackman et al., 2009; Petit et al., 2014).

Misplaced neurons in autistic brains often exhibit delayed maturation (Wegiel et al., 2010; Ben-Ari, 2014). *In utero* electroporation of mouse embryos with shRNAs against several neuronal migration-related genes, e.g., DCX and TBC1 domain family member 24 (TBC1D24), not only retarded radial migration of transfected neurons but also delayed maturation of ectopic neurons (Ackman et al., 2009; Falace et al., 2014; Martineau et al., 2018), presumably due to insufficient trophic support from the target area. Ectopic neurons lack several mature neuronal marker, but have immature dendritic arborization and spine formation, insufficient dendritic spine pruning, and delayed excitatory to inhibitory switch of GABAergic synaptic transmission (Wegiel et al., 2010). These are also important contributing factors of abnormal network activity in multiple brain disorders including ASD.

## Therapeutic Implication of Neuronal Migration Deficits in ASD

As a developmental disorder, the core symptoms of ASD emerge as a consequence of abnormal circuit wiring during embryonic development. One major challenge for ASD therapy is that it is not feasible to target the pharmacological intervention on embryonic developmental processes before the diagnoses of the disease. Moreover, special cautions need to be paid to the risk of unexpected side effects of any pharmacological agents on embryos. It is important, therefore, to explore therapeutic interventions that can effectively reverse the symptom after the diagnosis of the disease at postnatal stage. For ASD associated with neuronal migration deficits, therapies may be designed to restore neuronal migration.

Several studies of experimental treatment targeting neuronal migration in mice and rats have shown promising therapeutic effects. In one such study, *in utero* electroporation of embryos with shRNAs against Dcx in newborn cortical neurons in mouse or rat embryos was performed to generate subcortical band heterotopia. Electroporated animals displayed a reduced threshold of convulsant-induced seizure, and misplaced neurons were stimulated to migrate by re-expressing Dcx at early postnatal stage. Re-starting migration in this way restored both the patterning of neurons and the threshold of convulsant-induced seizure (Manent et al., 2009). However, this study did not address whether there is a causal relationship between the rescue of neuronal migration and the recovery of seizure threshold. Nevertheless, these findings suggest that disorders of neuronal migration may be treatable by re-engaging developmental programs to reduce the size of cortical malformations and to restore network activities. More sophisticated studies need to be conducted to clarify whether migration deficits caused by mutations of other genes can also be restored by rescuing the expression and function of the mutated genes postnatally. It is also necessary to determine the critical time window for effective restoration of neuronal migration and network activity by re-expressing the affected gene.

In a study using lissencephaly 1 (Lis1) gene heterozygous knockout (Lis1^±^) mice, investigators found that *in utero* knockdown of calpain by shRNA partially rescued defective cortical layering in Lis1^±^ offspring, and intra-peritoneal injection of the calpain inhibitor ALLN to pregnant Lis1^±^ dams suppressed apoptotic neuronal cell death and neuronal migration defects in mutant pups (Yamada et al., 2009). The same research group later reported that postnatal treatment of Lis1^±^ mice with a blood-brain-barrier permeable calpain inhibitor rescued defective neuronal migration and improved axonal transport, brain circuit formation, and animal behavioral performance (Toba et al., 2013). Furthermore, acute application of the calpain inhibitor ALLN in brain slices of Lis1^±^ mice restored spontaneous and miniature excitatory postsynaptic current (EPSC) frequencies to wild-type levels. This effect may be due to inhibition in the cleavage of the calpain substrate αII-spectrin although Lis1 protein levels were not restored (Sebe et al., 2013). These findings showed the possibility of postnatal application of pharmacological agents to treat neurological defects associated with neuronal migration deficits, although the causality between the rescue of neuronal migration, neuronal activity, and animal behavior remains to be investigated.

Pharmacological intervention of neuronal migration was conducted in an autistic model of coiled-coil protein associated with myosin II and DISC1 (CAMDI) gene knockout mice. CAMDI maintains the acetylation of α-tubulin in the centrosome by suppressing the activity of histone deacetylase 6 (HDAC6). In CAMDI knockout mice, hyperactivity of HDAC6 causes deacetylation of α-tubulin in the centrosome, resulting in aberrant migration of cortical neurons and abnormal behaviors of the animals. Cortical radial migration and psychiatric problems of CAMDI knockout mice were rescued by an inhibitor of HDAC6 (Fukuda et al., 2016). Further studies are needed to address the timing, quantity, and brain region specificity of the drugs for effective treatment (Fukuda and Yanagi, 2017).

Together, these findings showed that although the relationship between the rescue of neuronal migration and the recovery of brain function remains to be determined, neuronal migration can be a promising target for drug screening in ASD animal models and in tissue culture. It is conceivable that early intervention is essential during a critical time window of postnatal brain development, in which both neuronal migration and network function are still reversible.

## Conclusion

Clinical brain imaging, neuropathological analysis, and animal model studies have revealed neuronal migration deficits as a hallmark of autistic brains. Neuronal migration deficits are very likely a fundamental contributing factor for ASD, and are important biomarkers for ASD diagnosis and phenotypic readout in drug screening for ASD treatment. Rapid progress has been made in exploring ways for postnatal treatment to reverse neuronal migration and brain network defects in rodent models of neuronal migration disorders. There is an urgent need to elucidate the postnatal time window for restoring neuronal migration and to explore the therapeutic effect of correcting neuronal migration for ASD treatment.

## Author Contributions

X-BY conceived the idea. All authors wrote the manuscript, and read and approved the final manuscript.

## Conflict of Interest

The authors declare that the research was conducted in the absence of any commercial or financial relationships that could be construed as a potential conflict of interest.
